# Global analyses of TetR family transcriptional regulators in mycobacteria indicates conservation across species and diversity in regulated functions

**DOI:** 10.1186/s12864-015-1696-9

**Published:** 2015-06-27

**Authors:** Ricardo J. C. Balhana, Ashima Singla, Mahmudul Hasan Sikder, Mike Withers, Sharon L. Kendall

**Affiliations:** Department of Pathology and Pathogen Biology, The Royal Veterinary College, Royal College street, Camden, London, NW1 OTU UK; Department of Microbial and Cellular Sciences, Faculty of Health and Medical Sciences, University of Surrey, Stag Hill, Guildford, GU2 7XH UK; Indian Institute of Technology Kanpur, Kanpur, India; Department of Pharmacology, Faculty of Veterinary Science, Bangladesh Agricultural University, Mymensingh, 2202 Bangladesh

**Keywords:** TetR, Mycobacteria, Tuberculosis, Motif analysis, Gene regulation, Conservation analysis

## Abstract

**Background:**

Mycobacteria inhabit diverse niches and display high metabolic versatility. They can colonise both humans and animals and are also able to survive in the environment. In order to succeed, response to environmental cues via transcriptional regulation is required. In this study we focused on the TetR family of transcriptional regulators (TFTRs) in mycobacteria.

**Results:**

We used InterPro to classify the entire complement of transcriptional regulators in 10 mycobacterial species and these analyses showed that TFTRs are the most abundant family of regulators in all species. We identified those TFTRs that are conserved across all species analysed and those that are unique to the pathogens included in the analysis. We examined genomic contexts of 663 of the conserved TFTRs and observed that the majority of TFTRs are separated by 200 bp or less from divergently oriented genes. Analyses of divergent genes indicated that the TFTRs control diverse biochemical functions not limited to efflux pumps. TFTRs typically bind to palindromic motifs and we identified 11 highly significant novel motifs in the upstream regions of divergently oriented TFTRs. The C-terminal ligand binding domain from the TFTR complement in *M. tuberculosis* showed great diversity in amino acid sequence but with an overall architecture common to other TFTRs.

**Conclusion:**

This study suggests that mycobacteria depend on TFTRs for the transcriptional control of a number of metabolic functions yet the physiological role of the majority of these regulators remain unknown.

**Electronic supplementary material:**

The online version of this article (doi:10.1186/s12864-015-1696-9) contains supplementary material, which is available to authorized users.

## Background

TetR family transcriptional regulators (TFTRs) are common one-component prokaryotic signal transduction systems. This family of regulators contain a conserved helix turn helix (HTH) motif at the N-terminal DNA-binding end of the protein and a ligand binding pocket at the C-terminal end. TFTRs are often repressors and bind to DNA to prevent transcription in the absence of a ligand. The binding of an effector molecule at the C-terminal pocket causes structural changes in the protein resulting in the release of the regulator from the DNA.

TFTRs are present in a large number of bacterial genomes with soil dwelling bacteria encoding the highest numbers [1]. The sequences for more than 200,000 TFTRs are available in public databases and structures for almost 200 have been solved. The paradigm was first described in *Escherichia coli* and TetR, the founding member of the family, is a repressor that controls the expression of a divergently oriented efflux pump that transports tetracycline out of the cell [2]. Tetracycline binds to the C-terminal ligand pocket of the *E. coli* TetR to alleviate repression of the pump. In general, TFTRs are best known to bind small molecule ligands to control divergently oriented efflux pumps and, in addition to *E. coli* TetR, there are several good model systems including *Staphylococcus aureus* QacR [3].

Although the control of drug efflux is a much documented role for this family, as more TFTRs are characterised we are beginning to appreciate that efflux is just one of the diverse functions controlled by this family. The range of TFTR controlled functions include: carbon metabolism, nitrogen metabolism, co-factor metabolism, cell to cell signalling and cell division [1]. TFTRs that do not conform to the paradigm and act as activators [4–6], serve as global regulators [7], interact with peptide ligands [8] and even regulate enzyme activity post-translationally [9] are being described. These observations clearly suggest that there is still much to be learned about this ubiquitous family.

In this paper we use computational analyses to characterise mycobacterial TFTRs. Mycobacteria comprise some of the most important bacterial pathogens including the main causative agents of human and veterinary tuberculosis (*Mycobacterium tuberculosis* and *Mycobacterium bovis,* respectively). The exposure to a series of different conditions inside the host, most of which are hostile, and the presence of actively growing and dormant stages imply a key role for the regulation of gene expression in the success of these pathogens. In *M. tuberculosis*, TFTRs are involved in controlling the expression of genes required for carbon utilisation, *kstR, kstR2* and *mce3R* [10–12], branched chain amino acid catabolism, *bkaR* [13] and antibiotic resistance, *Rv3066, ethR* [14, 15].

We show that TFTRs are the most abundant family of HTH regulators in mycobacteria and as such the majority remain uncharacterised. We identify all the TFTRs in 10 mycobacterial species and assess the conservation of these genes across the mycobacteria. We define a set of TFTRs that are conserved across all species and those that are unique in those species that cause tuberculosis. It has been shown that genomic context is a reliable tool for predicting the genes regulated by TFTRs [16] and so we use context to predict the functions controlled by a sub-set of mycobacterial TFTRs. TFTRs typically bind to palindromic operators, and we use MEME [17] to identify regulatory motifs in the intergenic regions of the divergently oriented conserved mycobacterial TFTRs.

## Results and discussion

### TFTRs are the most abundant type of HTH DNA binding proteins in mycobacterial genomes

The majority of HTH-containing DNA binding proteins are sub-divided into families based on the structure and spatial arrangement of the helices [18]. InterPro [19] was used to identify the total complement of HTH DNA-binding proteins across 10 mycobacterial genomes (see Methods) and to classify the mycobacterial HTH proteins into their different families. The results, alongside the number of ORFs of each of the species are given in Table 1.

**Table 1:** The total number of HTH proteins (including TFTRs) in mycobacterial genomes

Organism	Number of HTH DNA binding proteins	Number of TFTRs (% of HTH)	Gene number	TFTRs as a % of ORFs
*Mycobacterium tuberculosis*	161	52 (32 %)	3999	1.3
*Mycobacterium bovis*	161	51 (32 %)	3920	1.3
*Mycobacterium bovis BCG*	160	51 (32 %)	3952	1.3
*Mycobacterium avium*	274	131 (48 %)	4910	2.7
*Mycobacterium avium paratuberculosis*	228	110 (48 %)	4350	2.5
*Mycobacterium marinum*	272	124 (46 %)	5424	2.3
*Mycobacterium ulcerans*	201	88 (44 %)	4160	2.1
*Mycobacterium leprae*	39	10 (26 %)	1605	0.6
*Mycobacterium gilvum*	328	129 (39 %)	5531	2.4
*Mycobacterium smegmatis*	514	160 (31 %)	6716	2.4

We identified a total of 2338 HTH DNA binding proteins across the 10 mycobacterial genomes. Of these 2338, 906 are TFTRs. For the mycobacterial species analysed, the number of HTH DNA-binding proteins increases with increasing number of ORFs. In general the soil-dwelling species such as *M. gilvum* and *M. smegmatis* have a larger number of ORFs and so might be expected to contain a larger number of HTH DNA binding proteins. If we compare *M. gilvum* with *M. marinum*, two mycobacteria with similar genome size but one soil dwelling and one adapted for survival in fish and amphibians, we see a reduction in the number of HTH DNA-binding proteins in the host adapted species (272 for *M. marinum* compared to 328 for *M. gilvum*) indicative of a reduction in diversity of the conditions within the intra-cellular environment.

**Fig. 1:**
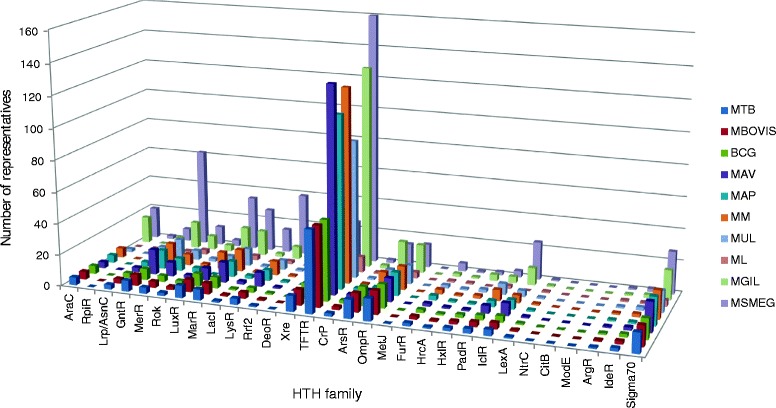
Numbers of HTH representatives in selected mycobacterial genomes grouped by family. The results were obtained by performing a search in the non-redundant proteome of each species using the Interpro signatures: AraC (IPR018060), RpiR (IPR000281), Lrp/AsnC (IPR000485), GntR (IPR000524), MerR (IPR000551), Rok (IPR000600), LuxR (IPR000792), MarR (IPR000835), LacI (IPR000843), LysR (IPR000847), Rrf2 (IPR000944), DeoR (IPR001034), Xre (IPR001387), TFTR (IPR001647), CrP (IPR001808), ArsR (IPR001845), OmpR (IPR001867), MetJ (IPR002084), FurR (IPR002481), HrcA (IPR002571), HxlR (IPR002577), PadR (IPR005149), IclR (IPR005471), LexA (IPR006199), NtrC (IPR010114), CitB (IPR012830), ModE (IPR016462), ArgR (IPR020900), IdeR (IPR022687), sigma 70 (IPR014284)

TFTRs make up 26–48 % of the HTH DNA-binding capacity in all species (Table 1, column 3 in brackets). In order to determine if the TFTRs were the most abundant type of HTH DNA-binding protein, the entire HTH complement across the 10 mycobacterial species was classified into family using InterPro. A complete list of genes belonging to each HTH family in all 10 genomes is given in Additional file 1: Table S1. The numbers of genes in each HTH family in 10 mycobacterial species are shown in Fig. 1. Within the HTH superclass, TFTRs are by far the most represented in all mycobacterial genomes. The next best represented HTH classes are GntR, enriched in *M. smegmatis* with 62 assignments but with a small number of representatives in the pathogenic mycobacteria, and OmpR – 14–15 members in all mycobacteria excluding *M. leprae*.

*M. leprae* has a drastically reduced genome and so a reduction in the number of TFTRs is expected. In order to determine whether the level of reduction in TFTRs was proportional to genome size we calculated the numbers of TFTRs as a percentage of open reading frames (Table 1, column 5). Interestingly, the percentage of TFTRs in the *M. leprae* was only 0.6 %, far less than the other mycobacteria possibly reflecting a disproportionate loss of this family in this species.

It is difficult to say whether mycobacterial genomes are enriched for TetR regulators from this analysis but by way of comparison, *E. coli* encodes 261 DNA-binding transcription factors in its 4.6 Mbp genome, of which only 5 % are TFTRs [1]. *Staphylococcus pyogenes*, another intra-cellular Gram positive pathogen, encodes approximately 81 DNA-binding factors, as part of its 1.85 Mbp genome, of which ~5 % are TFTRs. Soil dwelling bacteria are known to have a large number of TFTRs and so the large numbers in the pathogenic mycobacteria may be a reflection of their evolution from a soil dwelling ancestor [1].

### Conservation of TFTRs among the mycobacteria indicates a role in survival for both the environmental and pathogenic species

The advantage of assessing conservation at the genus level is that it might help to distinguish those TFTRs that are involved in shared processes from those that are required for the more adaptive functions. This is particularly important for mycobacteria where different species have different hosts in addition to environmental representatives. Conservation was assessed as described in the materials and methods. The results are given in Additional file 2: Table S2.

When *M. leprae* is included in the analysis, there are five TFTRs that are conserved across all mycobacteria analysed. These are shaded in blue in the Additional file 2: Table S2 (*Rv0238, Rv0472c, Rv3050c, Rv3208* and *Rv3855* (*ethR*)). The conservation of these regulators across all mycobacterial genomes, including the drastically reduced *M. leprae* genome suggests that the functions of these regulators are required for survival in both host adapted and environmental niches. The *M. leprae* gene *ML2457* is divergently oriented to a pseudogene and may not have a physiological role in this species. This group of regulators include *ethR*, a TFTR involved in antibiotic resistance that represses genes required for the activation of the antibiotic ethionamide. Mutations in this regulator cause resistance [15]. Its conservation in *M. smegmatis* and *M. gilvum* suggests that it might be useful in this species as a defence mechanism against antibiotic producers in the soil in the battle for resources.

Given that *M. leprae* has a much reduced genome and our previous analysis suggested a disproportionate loss of TFTRs we re-assessed conservation across mycobacterial genomes but this time excluded *M. leprae*. Those TFTRs that are conserved across all mycobacteria (excluding *M. leprae*) are shaded in green in Additional file 2: Table S2. The TFTRs present in *M. tuberculosis* are, in general well conserved with 22 of the 52 regulators having orthologs in all species included in the analysis. This group of regulators include *kstR* (*Rv3574*) and *kstR2* (*Rv3557c*), involved in cholesterol catabolism [10, 11, 20]*.* Their conservation in both pathogenic and environmental species suggests sterols are likely to be encountered in the environment (phytosterols and ergosterols) as well as in the host (host cholesterol). The conservation of the KstR regulators in *M. avium subspecies paratuberculosis* suggests that cholesterol catabolism is also important for this intestinal pathogen. This is supported by the recent observation that cholesterol is a carbon source for *M. avium subspecies paratuberculosis* in the bovine intestine [21].

### Conservation analysis identifies those TFTRs that are only present in the pathogenic representatives

In order to identify those TFTRs that might be uniquely involved in pathogenic processes (i.e. conserved in the pathogens but not conserved in the environmental species) we identified those TFTRs that were missing from both *M. smegmatis* and *M. gilvum* but present in the pathogenic species (Additional file 2: Table S2).

Only one regulator (Rv0078, shaded purple in Additional file 2: Table S2), was present in all pathogens, including *M. leprae.* However, the ortholog in *M. leprae* (*ML2677*) is divergently oriented to a pseudogene and so it is possible that it does not have a physiological role in *M. leprae*. Excluding the disproportionately reduced *M. leprae* from the analysis, three TFTRs (*Rv0653c, Rv1167c and Rv1556*) are conserved in the pathogenic species only and these are shaded in orange in the Additional file 2: Table S2. These candidates might control functions uniquely important for survival in the host.

Six genes were uniquely found in the species that cause tuberculosis (*Rv0302, Rv0328, Rv0330c, Rv1534, Rv2160A* and *Rv3160c*). These genes are shaded in red in Additional file 2: Table S2. With the exception of *Rv3160c* and *Rv2160A,* we currently do not have any experimental evidence of the functions that these six TFTRs might control. There is a frame shift mutation in *Rv2160A* in *M. tuberculosis* that makes it non-functional in this species. *Rv2160A* is situated on a likely operon with upstream and downstream genes *Rv2159c* and *Rv2161c,* respectively. These flanking genes show higher expression in *M. tuberculosis* and differential expression might have an impact on host preference [22]. *Rv2159c* is annotated as an alkyl hydro peroxidase, whereas *Rv2161c* is a conserved hypothetical protein. Their role in the physiology of the bacterium is unknown. *Rv3160c* and the neighbouring genes *Rv3161c* (a dioxygenase) and *Rv3162c* (a membrane protein) are induced upon exposure to antibiotics but the precise physiological functions of these genes remain unknown [23].

### The TFTR regulator *Rv1255c* is present in *M. tuberculosis* but missing from *M. bovis* and the vaccine strain *M. bovis* BCG Pasteur

The sequence of the *M. bovis* and *M. tuberculosis* genomes are 99.95 % similar and it has often been hypothesised that the widely different host preference exhibited by these species is a reflection of changes in gene expression rather than content. Aside from *Rv1255c*, the complement of TFTRs in *M. bovis* and *M. tuberculosis* is identical.

*Rv1255c* lies in the RD10 region which is part of a series of deletions that occurred in the “ancestral” – TbD1 + − species in the *Mycobacterium africanum* → *Mycobacterium microti* → *M. bovis* lineage. The RD10 deletion is present in strains that show wide host diversities and geography such as humans in Africa, voles in the UK, seals in Argentina, goats in Spain, and cattle and badgers in the UK [24, 25]. This regulator is on a putative two gene operon with the cytochrome p450 *cyp130* (*Rv1256c*), also within the RD10 region*.* Studies of the function and regulation of CYP130 in the “modern” – TbD1- strains of human adapted *M. tuberculosis* might allow us to gain additional knowledge of some of the biochemical differences between “modern” *M. tuberculosis* and “ancestral” and animal adapted species*.*

Similarly there are deletions in TFTRs in other strains of *M. bovis* BCG that might influence the efficacy of the vaccine. *Rv3405c* is in the RD16 region deleted from *M. bovis* BCG Moreau but a link between this deletion and vaccine efficacy is unknown [26].

### Most mycobacterial TFTR regulators are divergently oriented to an adjacent gene

It has been recently reported by Ahn *et al.,* that examination of the genome context of TFTRs can be a useful tool for the prediction of the genes they regulate [16]. This study, which focused on *Streptomyces,* showed that TFTRs that are divergently oriented to their neighbouring genes and separated by 200 bp or less can be reliably predicted to control the neighbouring gene. This analysis showed that the functions of the neighbouring gene(s) were more diverse than just drug efflux.

**Fig. 2:**
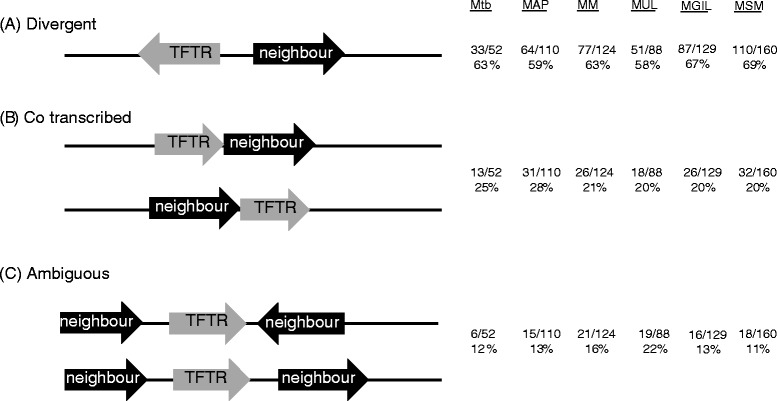
Classification of TFTRs according to relative orientation. 663 TFTRs from *M. tuberculosis* (MTB, 52 TFTRs), *M. avium subspecies paratuberculosis* (MAP, 110 TFTRs), *M. marinum* (MM, 124 TFTRs), *M. ulcerans* (MUL, 88 TFTRs), *M. gilvum* (MGIL, 129 TFTRs) and *M. smegmatis* (MSM, 160 TFTRs) were divided into three groups according to their genome context. **a** 408 TFTRs (33 in MTB, 64 in MAP, 77 in MM, 51 in MUL, 87 in MGIL and 110 in MSM) are encoded divergently to their neighbours. Here, the TFTR-encoding gene is located on the left side, but the positions of this gene and its divergent neighbour are interchangeable. **b** 146 TFTRs (13 in MTB, 31 in MAP, 26 in MM, 18 in MUL, 26 in MGIL and 32 in MSM) are likely co-transcribed with their upstream or downstream genes as the intergenic DNAs separating them are less than 35 bp. **c** 109 TFTRs (6 in MTB, 15 in MAP, 21 in MM, 19 in MUL, 16 in MGIL and 18 in MSM) show neither of the two aforementioned orientations

In order to examine the situation in mycobacteria, we analysed 663 TFTRs from *M. tuberculosis*, *M. avium paratuberculosis, M. marinum, M. ulcerans, M. gilvum* and *M. smegmatis*, for orientation, length of intergenic region and function of adjacent genes. The regulators were classified into groups **(A–C)** according to the criteria laid down by Ahn *et al.***(A)** divergent orientation with neighbour, **(B)** likely to be co-transcribed with upstream or downstream gene as they are in the same orientation and the intergenic DNA separating them is ≤ 35 bp, and **(C)** show neither **(A)** or **(B)**. The results are shown in Fig. 2.

In all six species approximately 60 % of the TFTRs are divergently oriented with their neighbour and this is similar to the figure reported by Ahn *et al.,* for *Streptomyces* species. The next most favoured arrangement is co-transcription with neighbouring genes followed by an ambiguous arrangement.

**Fig. 3:**
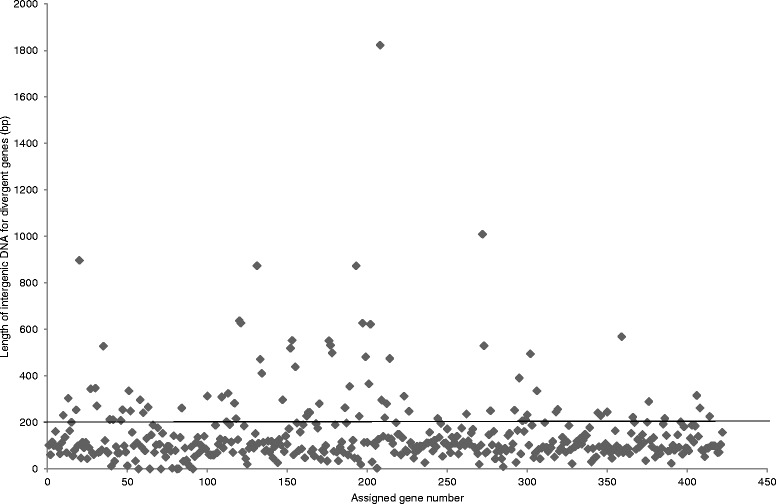
Lengths of the intergenic regions of the divergently oriented mycobacterial TFTR regulators. The intergenic regions from the 422 divergently oriented regulators from *M. tuberculosis* (Mtb), *M. avium paratuberculosis* (MAP), *M. marinum* (MM*), M. ulcerans* (MUL), *M. gilvum* (MGIL) and *M. smegmatis* (MSM) were analysed for length. Each dot represents an intergenic region and the length is given on the y-axis. Each of the genes were assigned a number e.g. 1–33 for MTB, 34–97 for MAP, 98–174 for MM, 175–225 for MUL, 226–312 for MGIL and 313–422 for MSMEG. The assignation of number was done in gene number order in each organism e.g. 1 = Rv0067c, 2 = Rv0078, 3 = Rv0135c etc. and this is given on the x-axis. The line represents a cut-off intergenic region size of 200 bp. The graph shows that the majority of divergently oriented genes are separated from their neighbour by 200 bp or less

For those that are divergently transcribed, the majority of regulators are separated from their divergent partners by 200 bp or less (Fig. 3). So, for *M. tuberculosis* 25 out of the 33 divergently oriented genes are separated by 200 bp or less (76 %) and such high frequencies are also observed in the rest of the mycobacteria (53/64 for *M. avium paratuberculosis* (83 %)*,* 58/77 for *M. marinum* (75 %), 34/51 for *M. ulcerans* (67 %), 74/87 for *M. gilvum* (85 %) and 96/110 for *M. smegmatis* (87 %). These analyses suggest that the majority of the divergently oriented TFTRs can be predicted to regulate the adjacent gene.

### Functional analysis of divergently oriented adjacent genes reveals that TFTRs control a diverse range of metabolic functions not limited to efflux

**Fig. 4:**
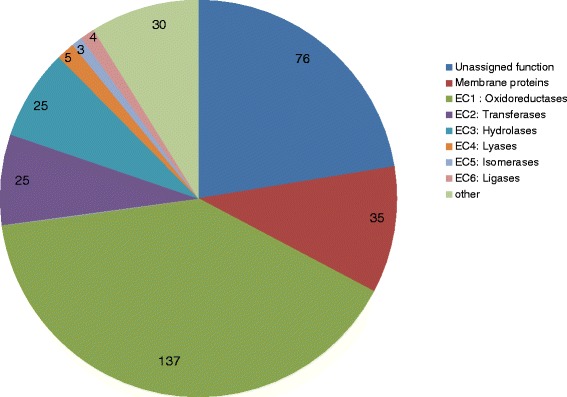
Functional classification of the products encoded by the divergent neighbouring genes. Genes that were divergently oriented to TFTRs with an intergenic region of 200 bp or less in *M. tuberculosis, M. marinum, M. avium paratuberculosis* and *M. smegmatis* were analysed as described in the materials and methods. Gene products that were enzymes were classified according to class (EC 1 to EC 6). Non enzymatic products were classified into membrane proteins, other proteins (e.g. transcriptional regulators), and proteins of unassigned function

We examined the functions of the genes divergent to the TFTRs in the six mycobacterial genomes in order to determine the possible functions regulated. We only included those genes that were separated from their divergent TFTRs by 200 bp or less. 340 genes from four different genomes (*M. tuberculosis, M. avium paratuberculosis, M. marinum M. ulcerans, M. gilvum* and *M. smegmatis)* were analysed in total. The results are shown in Fig. 4.

Fifty-eight percent of the divergently oriented genes are enzymes. The predicted enzymes were sub-divided into **E**nzyme **C**ommission (EC) number according to the reactions they were predicted to catalyse and by the presence of domains associated with that particular enzyme class. The majority of enzymes (40 %) are oxidoreductases (EC1) indicating that, in mycobacteria, the majority of TetR regulators control the expression of enzymes involved in energy and cellular metabolism, which may be crucial for metabolic adaptation.

Membrane proteins only account for 10 % of the functions of divergently oriented genes and attempts to further classify these were made using Pfam (http://pfam.xfam.org/) and Superfamily (http://supfam.cs.bris.ac.uk/SUPERFAMILY/). 22 of the 35 membrane proteins gave either no hits or contain a conserved domain of unknown function (pfam04286). 5 of the membrane proteins belong to the major facilitator superfamily of transporters (cl18950), 2 are PPE family proteins (pfam00823), 1 contains a mycobacterial membrane protein domain (pfam05423), 1 is a membrane bound histidine kinase (pfam00672), 1 is a chloride channel protein (pfam00654), 1 is a sodium decarboxylate symporter family (pfam00375), 1 is an ABC transporter family (pfam01061) and 1 is an amino acid permease (pfam13906).

These results are in agreement with the findings by Ahn *et al.* [16], and lend further support to the realisation that TFTRs do not just regulate efflux pumps. Our analyses suggest that TFTRs regulate a diverse range of as yet uncharacterised metabolic functions.

### Analysis of the upstream region of divergent TFTRs identifies 11 novel putative binding motifs

TFTRs typically bind to palindromic operators. The model TetR from *E. coli* binds as a dimer to a 15 bp palindrome while QacR from *S. aureus* binds as a tetramer to a 28 bp palindrome [3]. A number of TFTRs from *M. tuberculosis* (Rv3066, KstR, KstR2, BkaR) also bind to palindromic motifs [10, 11, 13, 14]. Motifs for Mce3R and EthR have also been described but these are larger, more complex, in multiple copies and do not conform to the classical structure of a palindromic sequence separated by a small number of bases [15, 27].

We used the programmes MEME and MAST to identify regulatory motifs in the intergenic regions for those regulators that were conserved across a number of species and were divergently oriented to the neighbouring gene [17]. A total number of 30 TFTRs were examined in the analysis, including the previously experimentally verified motifs. The results are given in Table 2.

**Table 2:** Motif analysis of the intergenic regions of conserved divergently oriented TFTRs

Gene number (name)	Motif	*e*-value
Rv2506 (bkaR)		1.20E–27
Rv3357c (kstR2)		4.40E–31
Rv3574 (kstR)		6.90E–36
Rv3855 (ethR)		8.90E–34
Rv0067c		1.10E–29
Rv0078		2.00E–36
Rv0158		5.60E–31
Rv0135c		6.00E–80
Rv0273c		1.20E–43
Rv0275c		9.00E–27
Rv0775		3.00E–34
Rv3055		1.80E–31
Rv3208		1.00E–25
Rv3405c		3.20E–28
Rv3830		6.20E–20
Rv0144		3.00E–16
Rv0452		4.90E–13
Rv0472c		5.60E–16
Rv0653c		1.10E–18
Rv0681		6.00E–17
Rv0691c		4.60E–02
Rv0767c		1.20E–14
Rv0825c		4.20E–14
Rv1019		1.90E–17
Rv1776c		4.00E–16
Rv2250c		2.40E–11
Rv3058c		2.00E–12
Rv3167c		4.20E–16
Rv3173c		3.00E–18
Rv3295		2.30E–16

The motif logo is given along with a significance estimate

The experimentally verified motifs show an e-value of > E-20 therefore we classified the motifs into highly significant (e value is = or > E-20) and less significant (<E-20). Of the 30 motifs that were analysed, 11 passed the cut-off. These represent a set of probable TetR binding motifs for the regulators listed.

### Conservation analysis of the C-terminal domain of TFTRs in *M. tuberculosis*

Although the work presented here and elsewhere support the idea that it is straightforward to predict at least one direct target gene for a previously unstudied TFTR, the real challenge is in the determination of the small-molecule ligands that the TFTRs bind to at the C-terminal end. Identification of the ligand is a means by which the biochemical function(s) of the target genes can be elucidated as many of the ligands bound by TFTRs are related to the biochemical functions of the target gene [28].

Previously phylogenetic analysis has been use to subdivide the GntR family of regulators in *M. tuberculosis* into functional clades based on the amino acid sequence similarity of their effector domain [29]. Additionally, phylogenetic approaches have been used to make general functional predictions for transcription factors for the AraC family [30]. Potentially a similar analysis could be applied to the C-terminal ligand binding domain of TFTRs to sub-divide the family into groups. Previously larger pan-genomic studies have grouped TFTRs based on amino acid sequence including TFTRs with known ligands and used this information to predict a ligand for a TFTR from Streptomyces, a prediction that was experimentally verified [28].

We aligned the C-terminus of all TFTRs in *M. tuberculosis* and attempted to establish phylogenetic groupings using widely-employed methodologies such as parsimony, maximum likelihood and neighbour joining. The alignments obtained were poor due to very low sequence similarity, approximately 7 % average identity between amino acid sequences. Conversely, the phylogenetic trees obtained (data not shown) showed overall weak groupings and no evident relationships. A previous study of the TFTRs reached a similar conclusion on the phylogeny of the C-terminus and found that the average identity score of the effector domain is only 9 % between TFTRs of known structure [31]. In contrast, an alignment of the N-terminal domain of the same TFTRs showed an average of 27 % identity. This reinforces the notion of a more conserved N-terminal (DNA binding) domain compared to a variable C-terminus.

Although amino acid sequences vary considerably, secondary structure prediction of the C-terminal ligand binding domain reveals conserved features. We predicted the secondary structures of each TFTR regulator in *M. tuberculosis* using JPred 3 and found a common architecture [32]. There are 6 α-helixes in the C-terminal ligand binding domain (α4 to α9) in most of the 52 regulators (Additional file 3: Figure S3). A few deletions seemed to have occurred, as in the case of α8 in one of the Mce3R heterodimers and Rv3066. Some insertions also occur Rv1353c (after α6) and Rv0330c (after α7). Although helixes are conserved in number, conservation of amino acid residues is extremely poor among the same helix for different regulators, with the exception of the first helix, α4, which produces a notably better alignment than the others. This could be expected considering that α4 directly interacts with the conserved HTH motif within the N-terminus and is part of the tetra-helical arrangement of the DNA binding region of TFTRs [18].

### Meta-analysis of published essentiality and expression studies triages those for further study and indicates infection relevant physiological functions for a selection of TFTRs

In order to determine those TFTRs that might have a role during infection in *M. tuberculosis* we performed a meta-analysis of selected published microarray studies to determine those TFTRs that are either essential or show expression changes in infection models or under *in vitro* conditions that mimic aspects of infection. The results of the analysis is shown in Additional file 4: Table S4.

Twenty-four TFTRs showed expression changes in at least 1 of the experimental conditions while 7 regulators were essential in at least one of the experimental conditions. This analysis helps to prioritise those TFTRs that might be taken forward for further study of the regulatory mechanisms involved in survival of *M. tuberculosis.*

Four regulators are essential for infection in the mouse model (*Rv2912c, Rv3050c, Rv3574* (kstR) and *Rv3855 (ethR)*). The physiological role of *kstR* is in the catabolism of cholesterol as a carbon source during infection [10, 20, 33], but the physiological role of the other essential TFTRs are unknown. The role of EthR in the control of *ethA*, an enzyme required for the activation of an anti-tuberculosis therapy ethionamide, is well documented but its physiological role remains unknown [15, 34–36]. Interestingly EthR is also induced under hypoxia and in dendritic cells. This analysis suggests an infection relevant physiological function for this regulator.

## Conclusion

TFTRs are especially frequent in organisms exposed to environmental alterations and stresses, for instance soil bacteria, and, not surprisingly, pathogenic species. Mycobacteria are a very versatile genus with species colonising very different environments, from soil dwelling saprophytic organisms, like *M. smegmatis* and *M. gilvum* to obligate human pathogens such as *M. tuberculosis* and *M. leprae* and also organisms that can coexist in both a parasitic and a free-living lifestyles such as *M. marinum* and *M.ulcerans*. To add to this inter-species versatility, the niche and lifestyle of each mycobacterial species can also be quite diverse, for example, *M. tuberculosis* has the ability to cause both life-threatening pulmonary tuberculosis and also clinically latent infections, living intra-cellularly as well as extra-cellularly and capable of infecting extra-pulmonary tissues. Such flexibility can only be achieved through changes in genetic expression, which is mostly mediated by transcriptional regulators.

In this work we have shown that the TFTRs are the most abundant family of transcriptional regulators with 906 TFTRs across the 10 species examined. Enrichment with such high numbers of TFTRs in mycobacterial genomes may be because TFTRs tend to control small regulons of neighbouring genes. Our data also suggests that mycobacterial TFTRs regulate divergent functions, including but extending beyond, efflux pumps. In fact, most mycobacterial TFTRs seem to be associated with enzymes which may reflect the metabolic plasticity in these species. Operator motif identification in mycobacteria is still in the early stages with motifs being identified for only a few transcriptional regulators [37]. We have identified 11 putative novel motifs for the TFTRs and these represent a set of sequences for testing. Only a few mycobacterial TFTRs have been well characterised to date, the importance of these in pathogenesis in *M. tuberculosis* or antibiotic resistance signifies that that further research into the uncharacterised TFTRs is necessary.

## Methods

### Identification and classification of the HTH DNA binding proteins in mycobacteria

The genome sequences of *Mycobacterium leprae (*NC_002677*)*, *Mycobacterium bovis* AF2122/97 *(*NC_002945*), Mycobacterium bovis* BCG Pasteur 1173P2 (NC_008769), *M. tuberculosis* H37Rv *(*NC_000962), *Mycobacterium avium subsp. paratuberculosis K-10 (*NC_002944) *Mycobacterium avium (*NC_008595*), Mycobacterium marinum (*NC_010612), *Mycobacterium ulcerans* (NC_005916), *Mycobacterium gilvum* (NC_009338) and *Mycobacterium smegmatis mc*^*2*^*155 (*NC_008596*)* were used in the analysis. These genomes represent those species that are obligate pathogens (*M. leprae*, *M. bovis* and *M. tuberculosis*), those that are able to cause disease but also survive outside the host (*M. avium*, *M. marinum M. avium subsp. paratuberculosis* and *M. ulcerans*) and those that are purely environmental (*M. smegmatis* and *M. gilvum*). The entire genome sequence from each species was searched using the integrated database Interpro in order to identify HTH DNA binding proteins and classify them into families.

### Conservation analysis of the TFTRs

Assessment of conservation and identification of orthologs was done using a combination of protein BLA*S*T, using the NCBI web server (http://www.ncbi.nlm.nih.gov/), and TB database [38] available at (http://tbdb.org/). Orthologs were identified by reciprocal protein-protein BLASTS (blastp algorithim). For example the amino acid sequence of a TFTR of one mycobacterium was compared to the entire ref-seq protein complement of the *M. tuberculosis* genome. The sequence of the most highly similar *M. tuberculosis* protein was used in another protein-protein BLAST against the original mycobacterium. Those genes that identified each other (i.e. reciprocal pairs) were considered potential orthologs. Pairwise identities were much less than 0.01 in each case and were more in the region of 1e–100. Amino acid sequence identities were greater than 50 % in each case over the entire length of the protein. Additionally, we used synteny as auxillary information for the detection or orthology. These were then checked using the TB database.

### Identification and functional analysis of adjacent genes

Genome context of the TFTRs in each genome was viewed using Artemis [39] available from the Web server **(**http://www.sanger.ac.uk/resources/software/artemis/). TFTRs were placed into their respective categories (A–C) and analysed in excel. For the divergently oriented genes the lengths of each of the intergenic regions were determined and only those genes that were separated by 200 bp or less were included in the functional analyses. In order to predict the functions of these divergently oriented genes, protein sequences were downloaded and a combination of protein BLAST, the TB database, and conserved domain search (CD) search was used (http://www.ncbi.nlm.nih.gov/Structure/cdd/wrpsb.cgi). Further analysis of membrane proteins was done using TMHMM [40] available at (http://www.cbs.dtu.dk/services/TMHMM/).

### Motif identification

Motif analysis was performed using MEME (http://meme-suite.org/) [17]. Intergenic regions from genes that were conserved among the mycobacteria and adjacently oriented genes were used in the analysis. This consisted of a group of 30 genes. The sequence of intergenic regions were extracted and uploaded in FASTA format. MEME was set to find palindromic motifs with a minimum width of 6 and a maximum of 50 bp. MEME was set to return a maximum of 3 motifs and the most significant motif was tabulated.

### C-terminal domain analyses

Multiple sequence alignment of TetR proteins was generated with Clustal software [41] available at (http://meme-suite.org/). The multiple alignments were either taken directly from the output generated by Clustal or manually improved based on secondary structure information using JPred 3 [32]. JPred 3 was used with the pre-set parameters available at (http://www.compbio.dundee.ac.uk/www-jpred/). Phylogenetic analyses were carried out using the PHYLIP software package. The SEQBOOT program was used to generate 1000 bootstrapping datasets for phylogeny estimations by parsimony and neighbour joining and 100 for maximum likelihood. Parsimony analysis was performed by running the multiple datasets through PROTPARS and CONSENSE, while neighbour joining analysis was done using PROTDIST, NEIGHBOUR and CONSENSE. Maximum likelihood analysis was carried out by inputting the 100 datasets in the program PROML and then running CONSENSE.

## Additional files

Additional file 1:
**Table S1**. Classification of all the HTH DNA binding proteins across 10 mycobacterial genomes. The first worksheet is the total number of representatives in each class for each genome. Each genome with the list of genes in each class are on separate worksheets. Each column in a worksheet represents a different HTH family with the family name and IPR number given in the column heading. Additional file 2:
**Table S2**. Conservation analysis of the mycobacterial TFTRs. Conservation was assessed relative to MTB. Genes that are conserved in all species including ML are shaded in blue. Those that are conserved across all mycobacteria with the exception of ML are shaded in green. Those that are conserved in all the pathogens (including ML) but missing from the non-pathogenic strains are shaded in purple and those that are conserved in all the pathogens (excluding ML) but missing from the non-pathogenic strains are shaded in orange. Those TFTRs conserved in those species causing tuberculosis are shaded in red. Additional file 3:
**Figure S3**. Secondary structure alignment of the C-terminus of the 52 TFTR regulators present in *M. tuberculosis* together with other regulators described previously in the literature. The structures were obtained using Jpred 3 and the each domain highlighted was aligned separately using ClustalX2. Additional file 4:
**Table S4**. Meta-analysis of microarray data. All 52 TFTRs from *M. tuberculosis* were examined for gene expression changes in key publications examining changes in gene expression and essentiality *in vivo* and *in vitro* under infection relevant conditions.

## Abbreviations

TFTRs: TetR family transcriptional regulators
HTH: Helix-turn-helix
EC: Enzyme commission, MTB, *Mycobacterium tuberculosis*
MBOVIS: *Mycobacterium bovis*
BCG: *Mycobacterium bovis BCG*
MAV: *Mycobacterium avium subspecies avium*
MAP: *Mycobacterium avium subspecies paratuberculosis*
MM: *Mycobacterium marinum*
MUL: *Mycobacterium ulcerans*
ML: *Mycobacterium leprae*
MGIL: *Mycobacterium gilvum*
MSM: *Mycobacterium smegmatis*
