# Supplementary figures and images for: Global analyses of TetR family transcriptional regulators in mycobacteria indicates conservation across species and diversity in regulated functions
Source: BMC Genomics. 2015 Jun 27;16(1):479. doi: 10.1186/s12864-015-1696-9 (PMC4482099; doi:10.1186/s12864-015-1696-9)

α9

α8

α7

α6

α5

α4


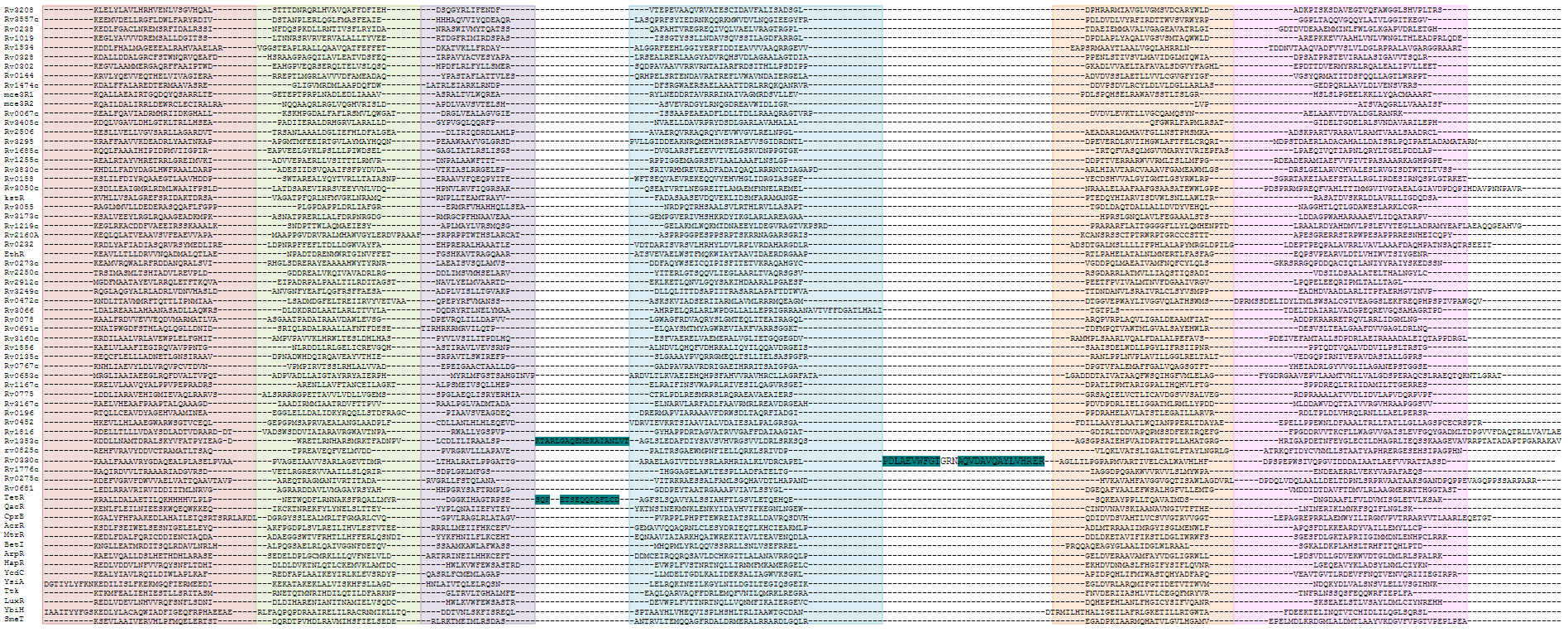

Supplement: Additional file 3: — Figure S3. Secondary structure alignment of the C-terminus of the 52 TFTR regulators present in M. tuberculosis together with other regulators described previously in the literature. The structures were obtained using Jpred 3 and the each domain highlighted was aligned separately using ClustalX2. [file 12864_2015_1696_MOESM3_ESM.docx]
